# Rapid early remission in a patient with severe aplastic anemia: a case report of hetrombopag, cyclosporine, and danazol combination therapy

**DOI:** 10.3389/fimmu.2025.1609771

**Published:** 2025-08-25

**Authors:** Yuting Dai, Qiang Guo, Xiaoxiao Guo, Qiuju Liu

**Affiliations:** Department of Hematology, Cancer Center, the First Hospital of Jilin University, Changchun, China

**Keywords:** severe aplastic anemia, case report, immunosupressive agents, antithymocyte globulin, cyclosporine, hetrombopag, danazol

## Abstract

Severe aplastic anemia (SAA) is a life-threatening bone marrow failure syndrome that is caused primarily by immune-mediated destruction of hematopoietic stem cells. Traditional treatment relies on immunosuppressive therapy (IST) with antithymocyte globulin (ATG) and cyclosporine (CSA). However, the toxicity and limited availability of ATG have spurred interest in ATG-free regimens. This case report describes a 28-year-old male with SAA who was treated with a combination of CSA, danazol, and hetrombopag (HPAG). The patient presented with pancytopenia and a hypocellular bone marrow, thus meeting the SAA criteria. He received CSA (5 mg/kg/day), HPAG (started at 10 mg and increased to 15 mg/day), and danazol (400 mg/day). Hematologic assessments using the NIH criteria revealed a partial response at 3 months and a complete response at 6 months, with reduced proportions of active T-cell subclones and no severe adverse events. This case suggests that the combination of CSA, HPAG, and danazol is effective in treating SAA, and a large-scale clinical trial is warranted to further confirm these promising results.

## Introduction

Severe aplastic anemia (SAA) is a life-threatening bone marrow failure syndrome that is characterized by pancytopenia and a hypocellular bone marrow. The pathogenesis of SAA is primarily immune mediated, with T-cell-mediated cytotoxicity playing a crucial role in the destruction of hematopoietic stem cells (HSCs) and the subsequent failure of normal hematopoiesis. According to the guidelines on SAA, for patients younger than 40 years who have an HLA-identical sibling donor, the preferred treatment is matched sibling donor hematopoietic stem cell transplantation (MSD-HSCT). For patients over 40 years of age who do not have HLA-compatible donors, the preferred first-line treatment is standard immunosuppressive therapy (horse-derived ATG and cyclosporin) ± a TPO-RA (1). The efficacy of eltrombopag (EPAG) addition was supported by landmark trials, such as the NIH single-arm study (2) and the European RACE trial (3), both reporting hematologic response rates of approximately 70–80%. Compared with horse ATG plus cyclosporin (CSA) alone, the combination of horse ATG, CSA and EPAG as a front-line therapy increased the overall response rate from 41% to 68% over the course of 6 months in SAA patients (3). However, rabbit ATG is often used when horse ATG is not available and has significantly inferior responses at 3 and 6 months and significantly shorter survival compared with those of horse ATG for first-line IST (at 6 months, the complete response rate was 3%, and the partial response rate was 37%) (4).

Furthermore, ATG therapy has many limitations, such as the high cost of the drug itself, as well as side effects that cannot be ignored, such as liver toxicity and serum sickness reactions. Moreover, ATG therapy must be administered at a hospital under the meticulous care of an experienced medical team. Therefore, the high cost of treatment and limitations in medical expertise prevent some patients from receiving standard IST therapy in economically disadvantaged and remote areas (5). In the real world, only 42.6% of patients receive AA-directed therapy for acquired AA, and triple therapy or HSCT is infrequently used for patients with heavily transfusion-dependent AA (HT-AA) (4.4% and 18.7%, respectively). The most common treatment is the combination of a calcineurin inhibitor (CNI) and eltrombopag (EPAG) (HT-AA, 51.7%) (6). Danazol, an androgen, not only promotes the secretion of EPO by the kidneys and increases the sensitivity of nucleated red blood cells to EPO but also increases telomere activity in human hematopoietic cells (7). It had already been used to treat AA before the emergence of ATG/CsA because it stimulates hematopoietic function. Therefore, danazol has usually been combined with cyclosporine to further improve the treatment efficacy.

In recent years, when the use of ATG is not feasible or its toxicity is intolerable, interest in the exploration of ATG-free treatment regimens has gradually increased. It is necessary to find more convenient and economical alternatives to ATG, especially in outpatient treatment for SAA. A recent study, the SOAR, indicated that the ATG-free regimen could be considered in SAA patients (8, 9). The SOAR trial, a multicenter, single-arm phase 2 study, assessed EPAG combined with CSA in treatment-naive adults with SAA. The overall hematologic response rate at 6 months was 46%. However, among patients who completed the 6-month treatment period, the primary endpoint of the overall response rate at 6 months was 63%, which was better than the outcomes of ITS containing rabbit ATG and CSA (4). For patients of Asian ethnicity (41%), the starting dose of EPAG was 100 mg/day. Moreover, the question remains whether it is feasible to increase the dose of TPO-RA in Asian populations and to combine the danazol as a new triplet in these SAA patients?

Herein, we report an Asian male patient with SAA who received a novel combination treatment with CSA, danazol, and hetrombopag (HPAG), demonstrating a rapid and effective response.

## Case presentation

**Figure 1:**
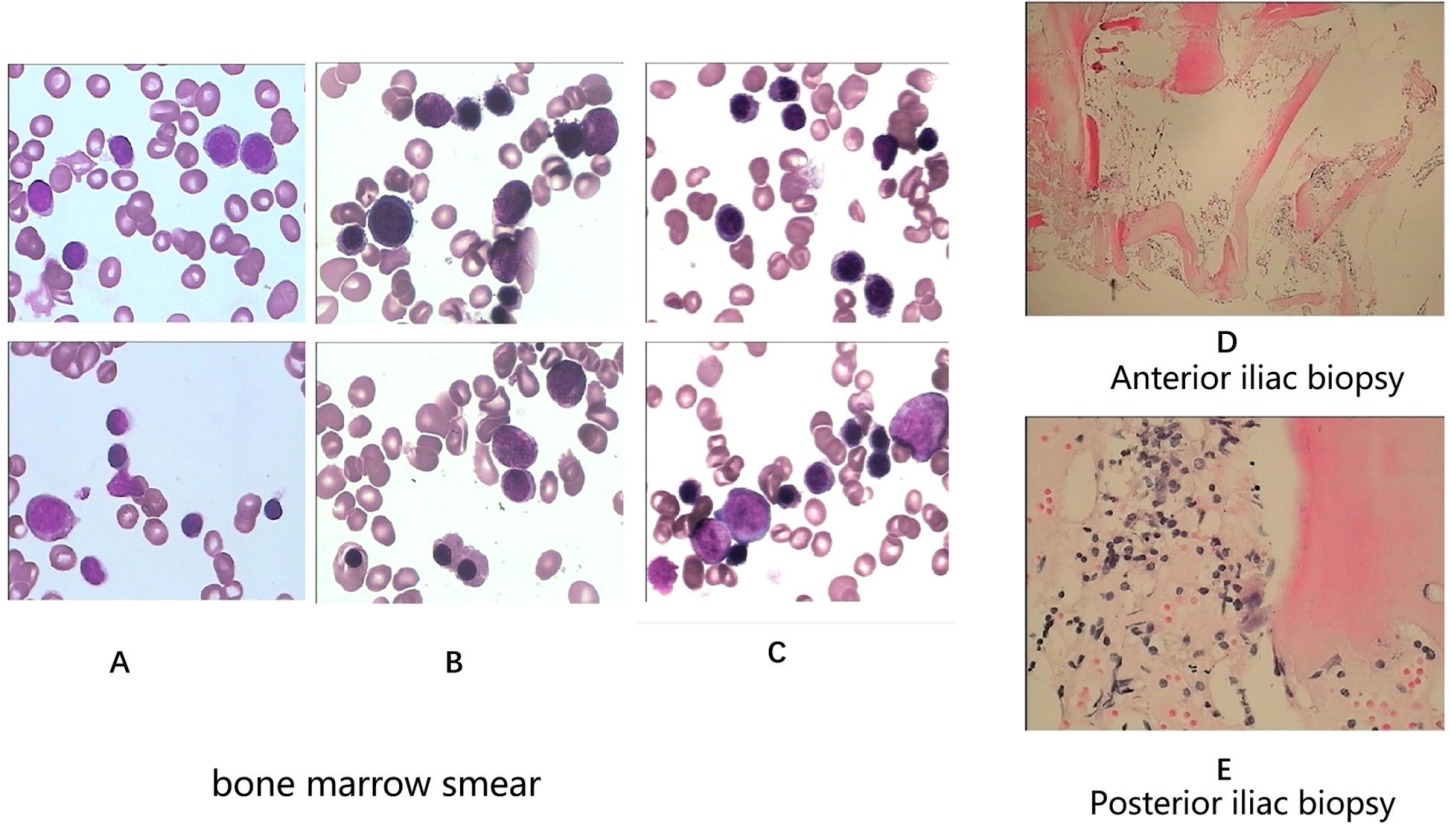
Bone marrow smears (**A**-anterior iliac bone, **B**-posterior iliac bone, **C**-sternum). Bone marrow biopsies (**D**-anterior iliac bone, **E**-posterior iliac bone) at initial diagnosis.

**Figure 2:**
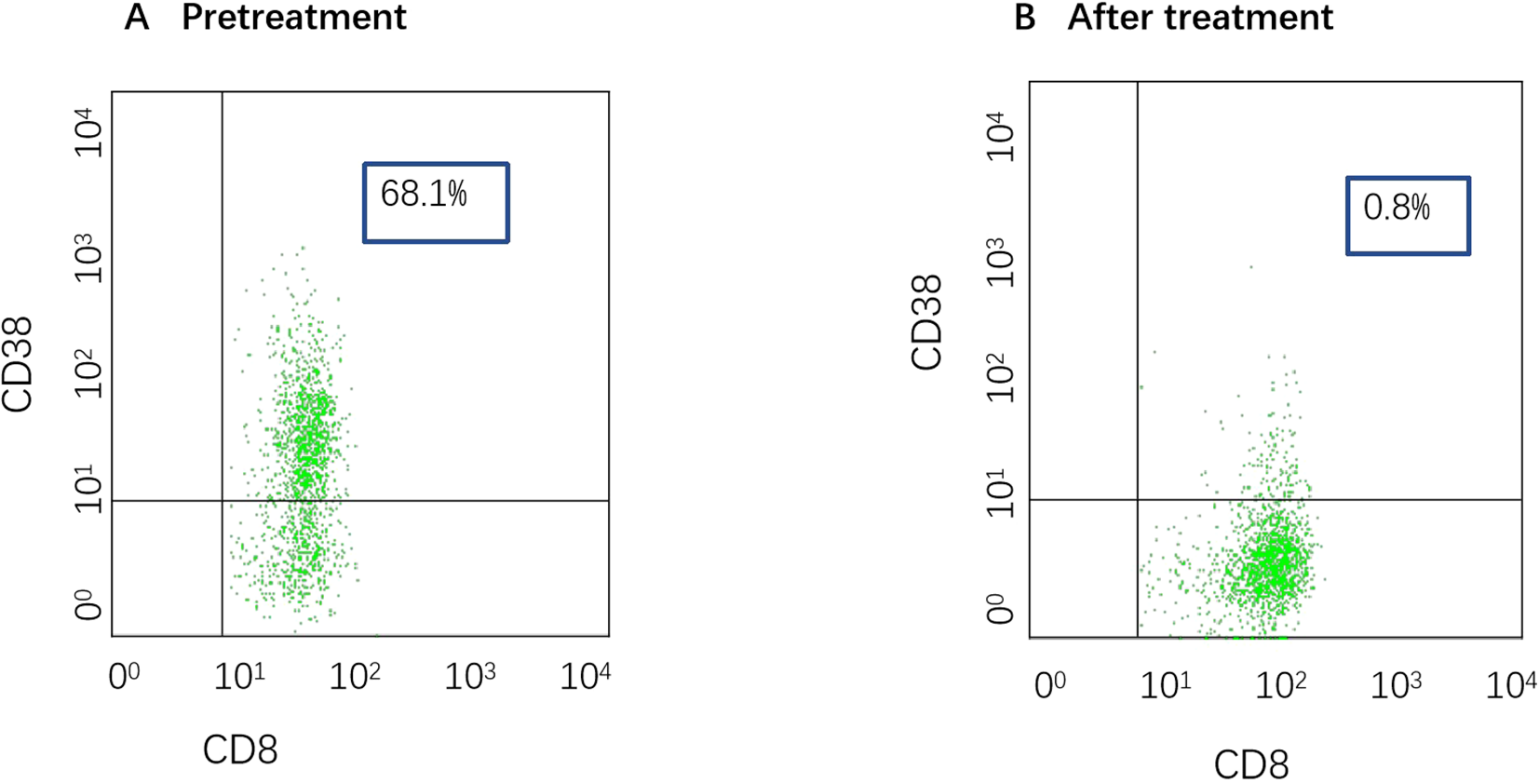
**(A)** T-cell subpopulation pretreatment (date: 2024.7.9). The proportion of activated CD8+CD38+ T cells was 68%. **(B)** T-cell subpopulation after treatment (date: 2025.3.26). The proportion of activated CD8+CD38+ T cells was 0.8%.

A 28-year-old male presented with sudden, unexplained fatigue that began approximately one month prior and progressively worsened despite rest. One week ago, he developed persistent gum bleeding, petechiae on his limbs, and black stools. He had no history of medication use or allergies. His past medical history included congenital cerebrovascular malformation for over 20 years, and but there was no significant family history of hematological disorders. On physical examination, he exhibited signs of anemia, scattered petechiae, and ecchymoses on his skin, with normal physical development and no evidence of deformity. Laboratory tests revealed hematologic parameters that were consistent with severe aplastic anemia (SAA) (1, 6), with bone marrow cellularity of less than 30%, an absolute neutrophil count (ANC) of 0.49×10^9^/L, a platelet count (PLT) of 7×10^9^/L, and a reticulocyte count (Ret) of 28.4×10^9^/L. Flow cytometry revealed no evidence of paroxysmal nocturnal hemoglobinuria (PNH). Liver, renal, and thyroid functions were normal, and autoimmune antibodies were negative. A lupus anticoagulant test, hemolysis test, antiphospholipid syndrome antibody tests, and ANA series tests were performed to screen for autoimmune disease, and the results were all negative. With respect to viral infections, tests for virus antibodies and nucleic acid quantification, including HCV, HBV, HIV, EBV, CMV, and B19 virus, were negative. Therefore, secondary blood cell decline caused by viral infection was excluded. Bone marrow examinations revealed no dysplasia or fibrosis. Marrow smears from three different sites (posterior iliac bone, anterior iliac bone and the sternum) revealed a decreased hematopoietic area with an absence of megakaryocytes. Biopsy of the iliac bone revealed low proliferation with a reduced hematopoietic area (**Figure 1**). The cytogenetic data were normal, and next-generation sequencing (NGS) analysis was negative. T-cell subclone analysis revealed that 68.1% (**Figure 2**) of active CD8+CD38+ T cells (activated T cells are functional T cells that are activated by costimulatory signals after antigen recognition) were positive for HLA-DR15.

**Figure 3:**
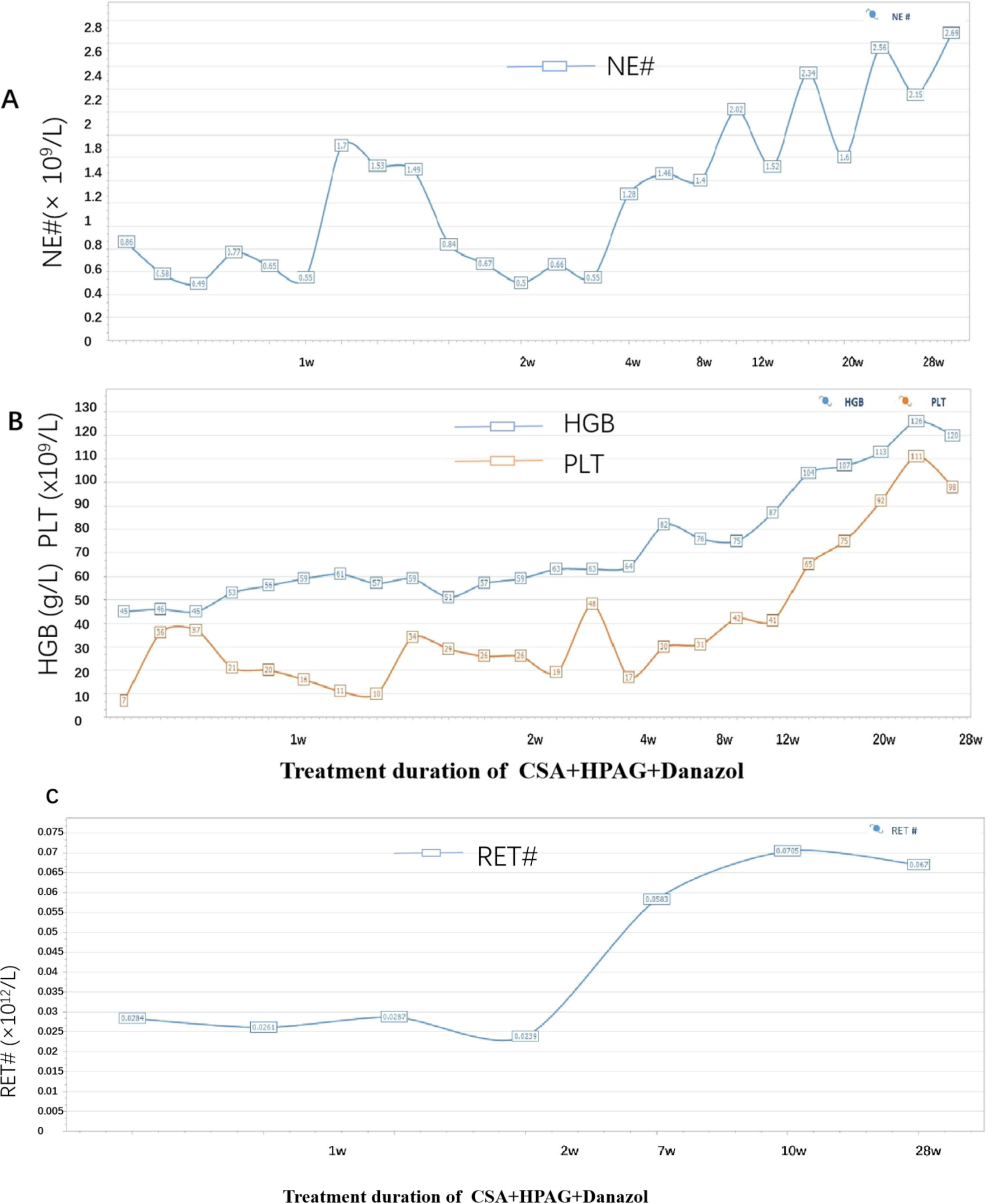
**(A)** Trend of changes in the absolute number of neutrophils. **(B)** Trend of changes in the hemoglobin and platelet levels (the blue line represents HGB, and the orange line represents PLT). **(C)** Trend of the absolute value of reticulocytes.

The patient had a matched sibling donor. However, owing to his economic conditions, the patient could not afford the cost of transplantation or the IST scheme containing ATG and requested that another scheme, with the lowest economic cost, be chosen. After carefully discussing the risks and benefits of HSCT, IST (ATG+CsA and HPAG) and HPAG+danazol+CsA, the patient was treated with a triple combination of CsA, danazol, and HPAG. CsA was initiated at 5 mg/kg/day with adjustment according to the serum drug concentration, targeting a plasma concentration of 200–400 ng/ml. HPAG was started orally at 10 mg per day and then increased to 15 mg 14 days later. Danazol was administered at 400 mg per day. Hematologic improvements were assessed using the National Institutes of Health (NIH) response criteria for SAA (9). At 3 and 6 months, hematologic evaluations revealed partial response (PR) and complete response (CR), respectively. At the 6-month follow-up, a CBC revealed an ANC of 4.02×10^9^/L, Hb of 135 g/L, a PLT of 127×10^9^/L, and a Ret of 670×10^9^/L. (**Figure 3** summarizes the hematologic parameters during treatment.) Liver and kidney functions were checked every two weeks, and the concentration of CSA was monitored monthly. The trough concentration of CSA fluctuated between 150 and 200 ng/ml, and there was no obvious liver or kidney impairment. T-cell subclone analysis revealed that the percentage of active CD8+CD38+ T cells was reduced to 0.8% (**Figure 2**). To date, the treatment process for over 1 year has been very favorable; no severe adverse effects have occurred, and no red blood cell or platelet transfusions have been needed for more than 9 months.

## Discussion

Traditional treatment strategies for SAA largely rely on immunosuppressive therapy (IST) with antithymocyte globulin (ATG) and CSA or hematopoietic stem cell transplantation (HSCT) for eligible patients. While CSA and EPAG are user friendly and generally safe, ATG not only has high production costs and high prices but also has toxicity to the heart and liver, increasing the risk of complications for older and weak patients. ATG also requires central venous catheterization and close monitoring of infusion reactions and cytokine release syndrome during medication. Standard IST treatment requires technical support from professional doctors and teams, and accessibility is limited (5, 8). Therefore, ATG availability is limited globally, necessitating the exploration of ATG-free regimens for certain regions and patients.

Preclinical and clinical data indicate that ATG-free regimens may be viable, with combinations of CSA and TPO-RAs, such as EPAG, showing promise in SAA treatment (8). The SOAR trial assessed the double combination of EPAG with CSA in treatment-naive adults with SAA. The overall hematologic response rate at 6 months was 46% (25 of 54 patients; 95% CI 33–60). Although the efficacy of the double combination was less than that of ATG-containing triple therapy (10, 11), its outcomes were not inferior to those of rabbit ATG. The common adverse events included elevated serum bilirubin (41%), nausea (30%), increased alanine aminotransferase levels (22%), and diarrhea (22%). No treatment-related deaths occurred. The results indicated that double therapy (CSA and EPAG) served as a well-tolerated, outpatient-first oral regimen for patients who could not obtain or tolerate ATG therapy. In this case, the patient’s limited financial resources made standard protocols, such as HSCT and IST, including ATG treatment, unfeasible. Therefore, exploring an effective yet cost-effective treatment plan, which is tailored to such patients, is essential. In this patient, we increased the dose of HPAG to 15 mg/d earlier and maintained a higher trough concentration of CSA (approximately 200 ng/ml) in combination with danazol as a third drug. This combination was novel and induced a favorable and rapid response in this patient.

Because of its hematopoietic stimulatory function, danazol was used to treat AA long before the advent of ATG and CSA. In some developing countries, androgens are still used as a first-line treatment for AA. Danazol is sometimes the only therapy for NSAA or MDS patients. The overall response rate of patients with AA to danazol alone can reach 40%, the CR rate is 27%, and the 5-year survival rate is 41%. In particular, the effect of increasing the platelet count is more significant (12). Another study treated 34 NSAA patients with CsA combined with danazol, and the overall response rate was 58.6% (13). A Chinese study revealed that the combination of CsA and androgens resulted in a hematological response in 68.8% of patients with non-SAA who relied on blood transfusions after 12 months of treatment (14). Factors such as age, disease duration, and androgen selection had minimal impacts on treatment effectiveness, whereas the platelet and reticulocyte counts were significant factors. The level of reticulocytes was associated with the hematological response at 12 months (14). Another study included 232 patients with SAA who received CsA and an LMS-based regimen containing CSA, levamisole, and danazol. The results revealed that patients who were younger than 40 years and had absolute neutrophil count (ANC)>0.2 × 10^9^/L, PLT>7 × 10^9^/L, and absolute reticulocyte count (ARC)>20 × 10^9^/L had longer survival and progression-free survival (*P <*0.001) *(* 15). Earlier research by Wang et al. highlighted synergistic effects among levamisole, CSA, and danazol in improving SAA outcomes (16). A Chinese multicenter study revealed that first-line treatment with CsA ± androgen + eltrombopag achieved high response rates compared with those of ATG-based regimens (HR 88.8% vs. 60% at 3 months) (17). Taken together, these studies indicate that ATG-free treatment might be possible in SAA patients.

However, the precise mechanism underlying the synergistic action between danazol and CSA remains unclear. One potential explanation is that CSA may significantly mitigate the immune response by inhibiting the production of negative factors such as interleukins (ILs) and tumor necrosis factor (TNF), thereby regulating the proportions of T-cell subsets and reducing immune-mediated attack on the hematopoietic system (18). Compared with those in healthy individuals, the proportions and function of regulatory T cells (Tregs) in the bone marrow and peripheral blood of patients with SAA are significantly lower, resulting in a loss of suppression of CD8+ T cells. The aberrant activation of CD8+ T cells leads to the destruction of hematopoietic stem cells, thereby contributing to the pathogenesis of the disease (19). Therefore, a targeted therapy to upregulate Tregs may benefit AA patients. However, CSA can inhibit the expression of IL-2 in T cells, thereby decreasing the number and function of Treg cells (20). In 2022, a report from China demonstrated that among patients with NSAA, the proportion of Tregs decreased in those treated with CSA, whereas it significantly increased in those treated with danazol. Despite the small sample size, these findings suggest that danazol positively impacts the quantity and function of Tregs. The use of danazol in combination therapy can counteract the suppressive effect of CSA on Tregs in NSAA patients, thereby enhancing immune tolerance while maintaining immunosuppressive efficacy (21). Therefore, the combination of CSA and danazol could enhance hematopoietic function.

In recent years, thrombopoietin receptor agonists (TPO-RAs), such as EPAG, have become increasingly necessary in the treatment of SAA (2, 22, 23). These drugs not only stimulate the proliferation and differentiation of megakaryocytes, thereby increasing platelet production, but also promote the survival and proliferation of hematopoietic stem cells. Additionally, these drugs exert immunomodulatory effects, regulating T- and B-cell functions and reducing the attack of hematopoietic stem cells by inflammatory factors. Both HPAG and EPAG can be used in patients with SAA. In the open-label EXTEND study, EPAG was associated with commonly reported adverse events, including cataracts (5%) and hepatobiliary adverse events (15%), with a thromboembolic event rate of 6% (24). HPAG, which is similar to EPAG but has less hepatotoxicity and a lower thrombotic risk, stimulates megakaryocyte proliferation via TPO-R activation, showing equivalent efficacy in patients with SAA (25–27). In China, HPAG is the only TPO-RA approved for the treatment of SAA by medical insurance. This advantage significantly reduces the financial burden of long-term treatment on patients. Another reason for selecting EPAG is the issue of racial dose limitations. In Asian populations, the maximum dosage of EPAG is 100 mg/day (equivalent to 10 mg/day of HPAG), whereas the maximum dosage of HPAG is 15 mg/day. Danazol, a synthetic androgen, promotes hematopoiesis, whereas CSA mitigates immune-mediated HSC destruction. As observed in our case, adding androgens such as danazol may maximize blood cell production in SAA patients, and this combination therapy offers a promising ATG-free alternative, warranting further evaluation in a larger phase II study and likely a phase III study to confirm its efficacy and safety.

In this case, the patient was treated with a triplet regimen comprising CSA, danazol, and HPAG. Within six months, hematologic evaluations demonstrated a complete response (CR), with platelet counts normalizing. This treatment is particularly suitable for patients because of its cost-effectiveness and outpatient administration, thereby alleviating the financial burden. While IST with ATG is effective for treating SAA, alternative therapies become crucial when patients are unsuitable for ATG or HSCT. In patients with a short disease duration, moderately low reticulocyte counts, residual hematopoiesis, financial constraints that preclude transplantation or IST, and no significant hepatic or renal dysfunction or other complications, a regimen of CSA combined with EPAG and danazol can be considered. In this context, adequate dosing of HPAG and maintaining a higher trough concentration of CSA are crucial. Our case report underscores the feasibility of this approach in non-VSAA patients with milder aplastic anemia, suggesting that ATG-free regimens combined with TPO-RAs may serve as viable alternatives for SAA patients.

## Data Availability

The original contributions presented in the study are included in the article/supplementary material. Further inquiries can be directed to the corresponding author/s.
